# Investigating dose-response patterns in virtual reality rehabilitation: a pilot study of patient satisfaction in subacute stroke

**DOI:** 10.3389/fresc.2025.1678042

**Published:** 2025-10-16

**Authors:** Iva Fiedorova, Sarka Banikova, Alice Najsrova, Istvan Szegedi, Katerina Vitova, Jana Trda, Ondrej Volny

**Affiliations:** ^1^ Department of Rehabilitation and Sports Medicine, University Hospital Ostrava, Ostrava, Czechia; ^2^ Department of Rehabilitation and Sports Medicine, Faculty of Medicine, University of Ostrava, Ostrava, Czechia; ^3^ Centre for Clinical Neurosciences, Faculty of Medicine, University of Ostrava, Ostrava, Czechia; ^4^ Department of Neurology, University Hospital Ostrava, Ostrava, Czechia; ^5^ Department of Neurology, University of Debrecen, Debrecen, Hungary; ^6^ VR LIFE Ltd., Ostrava, Czechia

**Keywords:** virtual reality, stroke rehabilitation, dosage, patient satisfaction, neurorehabilitation

## Abstract

**Background:**

Virtual reality (VR) rehabilitation shows promise for stroke recovery, but optimal dosage remains unclear. We examined the relationship between VR therapy intensity and patient satisfaction, while assessing methodological challenges in dose-response research.

**Objective:**

To investigate relationships between VR rehabilitation dosage (sessions, duration) and patient satisfaction in subacute stroke patients, and identify requirements for future definitive studies.

**Methods:**

We analyzed data from 19 subacute ischemic stroke patients who received VR rehabilitation using VR Vitalis® Pro system (January–December 2024). Patient satisfaction was measured with the User Satisfaction Evaluation Questionnaire (USEQ). We examined correlations between VR dosage variables and satisfaction, then conducted *post-hoc* power analysis and confounding assessment.

**Results:**

Patients averaged 25.0 ± 6.8 USEQ points, with 68% achieving high satisfaction. They completed 4.2 ± 4.1 VR sessions (range 1–13), but 58% received only 1–2 sessions due to clinical factors. No significant correlation emerged between sessions and satisfaction (*r* = 0.18, *p* = 0.47). *Post-hoc* analysis revealed only 11% statistical power for the observed effect. VR sessions strongly correlated with hospital stay (*r* = 0.664, *p* = 0.002), indicating confounding by clinical severity rather than research-controlled dosage.

**Conclusions:**

Our underpowered study (11% power) with substantial clinical confounding cannot determine dose-response relationships or inform practice. Future studies need larger samples (*n* ≥ 85) with randomized dosage allocation. Our main contribution is demonstrating methodological requirements for rigorous VR dose-response research.

## Introduction

Virtual reality (VR) rehabilitation has gained recognition as an effective adjunctive therapy for stroke recovery, with meta-analyses demonstrating benefits for motor function, balance, and cognitive outcomes (1, 2). However, despite growing evidence for VR efficacy, clinical implementation remains challenged by lack of standardized dosage protocols. The question of “how much VR is enough?” represents a critical gap in translating research findings to clinical practice.

Traditional rehabilitation research has established dose-response relationships for conventional therapies, where increased therapy intensity generally correlates with improved outcomes (3). However, VR rehabilitation presents unique considerations that may alter this relationship. VR therapy can be implemented through different levels of immersion: fully immersive (complete integration using head-mounted displays), semi-immersive (large projection surfaces without head-mounted displays), or non-immersive approaches (traditional computer interfaces). The neurobiological foundation for VR neurorehabilitation lies in mirror neuron activation across motor-related brain regions, leading to improved interhemispheric balance and enhanced neuroplasticity. For comprehensive review of VR mechanisms and clinical applications in stroke rehabilitation, we refer readers to our detailed analysis published elsewhere (4). The immersive, engaging nature of VR can potentially achieve therapeutic gains with shorter exposure times compared to conventional exercises (5). Additionally, patient tolerance factors such as cybersickness, visual fatigue, or cognitive overload may create upper limits on optimal VR dosage (6).

Previous VR rehabilitation studies have employed widely varying protocols, from single 20 min sessions to daily 60 min interventions over several weeks (7, 8). This heterogeneity makes it difficult to establish evidence-based dosage recommendations. Furthermore, most studies focus on clinical effectiveness rather than patient-centered outcomes such as satisfaction and acceptance, which are crucial for long-term adherence and real-world implementation success.

Patient satisfaction serves as an important indicator of treatment acceptability and may predict engagement with rehabilitation programs (9). The User Satisfaction Evaluation Questionnaire (USEQ) provides a validated framework for assessing patient experiences with virtual rehabilitation systems, examining key dimensions including enjoyment, perceived success, system control, information clarity, comfort, and perceived therapeutic benefit (10). Understanding this relationship could inform optimal therapy prescriptions and help clinicians balance therapeutic intensity with patient tolerance, building upon our demonstrated high satisfaction rates (68.4% achieving high USEQ satisfaction) and good clinical outcomes in this patient population (11).

The primary objective of this secondary analysis was to examine correlations between VR rehabilitation dosage parameters (number of sessions, session duration, cumulative exposure time) and patient satisfaction scores in subacute ischemic stroke patients. Secondary objectives included identifying potential threshold effects and exploring individual factors that may moderate dose-response relationships.

## Methods

### Study design and participants

This secondary analysis utilized data from our prospective pilot study conducted from January 1 to December 31, 2024, at University Hospital Ostrava, Czech Republic. The original study protocol was approved by the institutional ethics committee (University Hospital Ostrava, No. 766/2023). Detailed inclusion and exclusion criteria have been previously published (11).

Briefly, participants were adults (≥18 years) with ischemic stroke confirmed by neuroimaging, in the subacute phase (≤2 weeks post-stroke), with stable medical condition suitable for rehabilitation and sufficient cognitive function to understand VR instructions. Exclusion criteria included hemorrhagic stroke, severe cognitive impairment, history of epilepsy, severe uncorrectable visual impairment, motion sickness disorders, and unstable cardiovascular condition.

### VR rehabilitation intervention

All patients received VR rehabilitation using the MDR-certified VR Vitalis® Pro system (VR LIFE Ltd., Ostrava, Czech Republic) utilizing Oculus Quest 2 VR headsets with wireless controllers for both upper and lower limb exercises. The VR program included bilateral upper limb coordination exercises (“Hanging laundry”), reach-and-grasp activities (“Carrying mugs to shelves”), balance training, and cognitive-motor dual tasks. Each session lasted approximately 10–20 min, with frequency and content individualized based on patient tolerance. VR sessions were initiated at mean 6 days post-stroke, provided once daily when clinically appropriate based on patient tolerance, and medical stability. Session duration was automatically recorded by the VR Vitalis® Pro system. For comprehensive review of VR principles and mechanisms in stroke rehabilitation, we refer readers to our detailed analysis (4).

### Dosage variables

For this analysis, VR dosage was quantified using three primary variables:

1. **Total number of VR sessions** completed during the rehabilitation course
2. **Average session duration** (minutes per session)
3. **Cumulative VR exposure time** (total minutes across all sessions)

Additional variables included days from stroke onset to VR initiation and total rehabilitation duration.

### Outcome measures

Patient satisfaction was assessed using the USEQ, a validated 6-item instrument with 5-point Likert scales (1 = not at all, 5 = very much). The questionnaire addresses enjoyment, perceived success, system control, information clarity, comfort, and perceived rehabilitation benefit. Total scores range from 6 to 30 points, with ≥25 indicating high satisfaction, 15–24 medium satisfaction, and <15 low satisfaction. One item (Q5: discomfort) is reverse-scored for total calculation (10). The USEQ was administered at hospital discharge, after completion of all VR rehabilitation sessions, by trained rehabilitation staff. This timing ensured that patients could evaluate their overall VR rehabilitation experience rather than satisfaction with individual sessions.

### Statistical analysis

Descriptive statistics summarized patient characteristics and dosage variables. Continuous variables are presented as mean ± standard deviation or median (interquartile range) as appropriate. Categorical variables are presented as frequencies and percentages.

Correlation analyses examined relationships between VR dosage parameters and USEQ scores using Pearson correlation coefficients for normally distributed variables and Spearman correlations for non-parametric data. Individual USEQ item responses were analyzed to identify specific satisfaction domains most sensitive to dosage effects.

Patients were stratified by VR session number (<5 sessions vs. ≥5 sessions) and session duration (<10 min vs. ≥10 min) to explore potential threshold effects. The stratification cut-off points were chosen *post-hoc* based on data distribution rather than established evidence-based thresholds. Between-group comparisons used independent t-tests or Mann–Whitney *U*-tests as appropriate. Statistical significance was set at *p* < 0.05.

We conducted *post-hoc* power analysis to determine the ability to detect clinically meaningful correlations (effect sizes *r* = 0.1–0.6) and calculated required sample sizes for adequate power. We also examined potential confounding by analyzing correlations between VR dosage and hospital length of stay, since VR exposure was clinically determined rather than randomized. The distribution of VR sessions across patients was characterized to assess coverage of different dosage levels. Given the small sample size and non-randomized dosage allocation, all analyses were considered exploratory rather than confirmatory.

## Results

### Patient characteristics and VR dosage

Nineteen patients completed VR rehabilitation and satisfaction assessments. Mean age was 67.7 ± 11.2 years with balanced gender distribution (52.6% female). All patients had ischemic stroke, and VR rehabilitation was initiated at mean 6 days post-stroke.

VR dosage showed considerable variability across patients (Table 1). Total VR sessions ranged from 1 to 13 per patient (mean 4.2 ± 4.1). Average session duration was 12.8 ± 4.5 min (range 7–20 min). Cumulative VR exposure time ranged from 7 to 208 min (mean 58.1 ± 48.3 min). This variability reflected individualized protocols based on clinical needs, tolerance, and length of hospital stay.

**Table 1:** Patient characteristics and VR dosage parameters.

Variable	Value
Demographics	
Age, mean ± SD (range), years	67.7 ± 11.2 (46–86)
Female, *n* (%)	10 (52.6)
VR Dosage Parameters	
Total VR sessions, mean ± SD (range)	4.2 ± 4.1 (1–13)
Average session duration, mean ± SD (range), min	12.8 ± 4.5 (7–20)
Cumulative VR time, mean ± SD (range), min	58.1 ± 48.3 (7–208)
VR initiation post-stroke, mean ± SD, days	6.0 ± 3.2

### Satisfaction outcomes

Mean USEQ satisfaction score was 25.0 ± 6.8 points (range 7–30). High satisfaction (≥25 points) was achieved by 13 patients (68.4%), medium satisfaction (15–24 points) by 5 patients (26.3%), and low satisfaction (<15 points) by 1 patient (5.3%). Individual USEQ items showed highest ratings for information clarity (4.63 ± 0.96) and perceived rehabilitation benefit (4.37 ± 1.12).

### Dose-Response relationships

Correlation analyses revealed no significant relationships between VR dosage parameters and overall USEQ satisfaction scores (Table 2). Total number of VR sessions showed weak positive correlation with USEQ scores (*r* = 0.18, *p* = 0.47). Average session duration demonstrated minimal correlation (*r* = 0.12, *p* = 0.63), while cumulative VR time showed similarly weak association (*r* = 0.21, *p* = 0.39).

**Table 2:** Correlation analysis between VR dosage parameters and USEQ items.

USEQ item	Total VR sessions	Average session duration (min)	Cumulative VR time (min)
Q1: Enjoyment	*r* = 0.24, *p* = 0.32	*r* = 0.15, *p* = 0.54	*r* = 0.28, *p* = 0.24
Q2: Success	*r* = 0.12, *p* = 0.63	*r* = 0.08, *p* = 0.74	*r* = 0.16, *p* = 0.52
Q3: Control	*r* = 0.19, *p* = 0.44	*r* = 0.28, *p* = 0.24	*r* = 0.24, *p* = 0.32
Q4: Information	*r* = 0.06, *p* = 0.81	*r* = 0.11, *p* = 0.66	*r* = 0.09, *p* = 0.71
Q5: Comfort*	*r* = 0.09, *p* = 0.71	*r* = −0.03, *p* = 0.90	*r* = 0.11, *p* = 0.65
Q6: Benefit	*r* = 0.15, *p* = 0.54	*r* = 0.31, *p* = 0.19	*r* = 0.22, *p* = 0.36
Total USEQ score	***r* = 0.18, *p* = 0.47**	***r* = 0.12, *p* = 0.63**	***r* = 0.21, *p* = 0.39**

* All correlations non-significant at *p* < 0.05 level. Q5 analyzed as original scores (higher = more discomfort). Study was underpowered to detect small-to-medium effect sizes.

Analysis of individual USEQ items revealed more nuanced relationships. Session duration showed modest positive correlation with perceived rehabilitation benefit (Q6: *r* = 0.31, *p* = 0.19) and system control (Q3: *r* = 0.28, *p* = 0.24), though these did not reach statistical significance. Number of sessions correlated weakly with enjoyment ratings (Q1: *r* = 0.24, *p* = 0.32).

### Threshold analysis

Patients receiving ≥5 VR sessions (*n* = 9) achieved numerically higher rates of high satisfaction compared to those receiving <5 sessions (*n* = 10): 77.8% vs. 60.0%, respectively (*p* = 0.43). Mean USEQ scores were similar between groups (26.1 ± 5.8 vs. 24.0 ± 7.6 points, *p* = 0.48).

Session duration stratification showed patients with ≥10 min sessions (*n* = 15) had slightly higher satisfaction than those with <10 min sessions (*n* = 4): mean USEQ 25.4 ± 6.9 vs. 23.5 ± 6.8 points (*p* = 0.62).

### Safety and tolerance

Importantly, no significant correlations were found between VR dosage and reported discomfort levels (Q5 reverse-scored: *r* = −0.03 to 0.09 across dosage variables). This suggests that increased VR exposure within the studied range did not compromise patient tolerance or safety.

### *Post-hoc* power analysis and confounding assessment

*Post-hoc* power analysis revealed limited statistical power with our sample size (*n* = 19). The study had approximately 20% power to detect small correlations (*r* = 0.1–0.2) and 20%–40% power to detect medium correlations (*r* = 0.3–0.4), well below the conventional 80% threshold. To detect a clinically meaningful correlation of *r* = 0.3 with 80% power would require approximately 92 patients.

Analysis revealed significant confounding by clinical factors. Hospital length of stay showed strong positive correlation with number of VR sessions (*r* = 0.664, *p* = 0.002), indicating that VR dosage was primarily determined by stroke severity and medical stability rather than research-controlled parameters. Patients with longer hospital stays systematically received more VR sessions due to extended rehabilitation needs. The distribution of VR sessions was highly uneven: 6 patients (31.6%) received 1 session, 5 patients (26.3%) received 2 sessions, 2 patients (10.5%) received 3–5 sessions, 4 patients (21.1%) received 6–10 sessions, and 2 patients (10.5%) received >10 sessions. This distribution pattern reflected clinical decision-making rather than systematic dosage allocation. Confounding Analysis and VR Dosage Distribution is presented in Tables 3A,B.

**Table 3:** Confounding analysis and VR dosage distribution.

(A) Potential confounding variables:				
Variable	Correlation with VR sessions		*p*-value	Clinical interpretation
**Hospital length of stay**	***r* = 0.664**		***p* = 0.002**	**Strong confounder**
Age	*r* = −0.18		*p* = 0.46	Minimal confounding
Days to VR initiation	*r* = 0.31		*p* = 0.20	Moderate confounding
(B) VR session distribution (showing “scattered range”):				
Sessions	*n* (%)	Cumulative %	Mean USEQ ± SD	High satisfaction*
1	6 (31.6%)	31.6%	24.2 ± 8.1	4/6 (66.7%)
2	5 (26.3%)	57.9%	23.8 ± 7.2	3/5 (60.0%)
3–5	2 (10.5%)	68.4%	21.0 ± 4.2	1/2 (50.0%)
6–10	4 (21.1%)	89.5%	27.5 ± 3.1	3/4 (75.0%)
11–13	2 (10.5%)	100%	26.0 ± 0.0	2/2 (100%)

* High satisfaction = USEQ ≥25 points.

## Discussion

The most critical issue we need to address is our study's limited statistical power. With only 19 patients, we had just 8%–23% power to detect small-to-medium correlations (*r* = 0.1–0.3) that could be clinically important in rehabilitation research. When we found no significant dose-response relationships between VR therapy intensity and patient satisfaction, this doesn't necessarily mean such relationships don't exist—we simply couldn't detect them with our sample size.

Looking back at our data, we discovered following. The number of VR sessions patients received was strongly tied to how long they stayed in hospital, which likely reflects stroke severity more than any treatment decision we could control. Most patients (58%) got only 1–2 VR sessions because of early discharge, not because we planned it that way. This means our “dosage” variable was really just a marker of clinical severity rather than something we could meaningfully study. This creates a fundamental confounding problem that makes our correlation analysis questionable. Patients who received more VR sessions were systematically different—they had longer hospital stays that reflected more severe strokes or slower recoveries. We can't separate potential VR dosage effects from these underlying clinical differences, making any causal interpretation inappropriate.

The absence of significant correlations cannot be taken as evidence that dose-response relationships don't exist in VR rehabilitation. A true relationship may well be there but remain hidden in our small, confounded dataset. The trends we observed—like slightly higher satisfaction in patients receiving 5 + sessions (77.8% vs. 60.0% achieving high satisfaction)—are intriguing but could easily be chance findings given our limited power.

While we can't draw firm conclusions, our results do raise some interesting questions worth exploring in future research. The lack of strong dose-response patterns could potentially support findings by Karamians et al. suggesting that VR's interactive elements create enhanced motivation compared to conventional exercises. The immersive, engaging nature of VR might achieve high patient satisfaction even with brief exposures, unlike traditional rehabilitation where increased therapy intensity typically correlates with improved outcomes. The novelty and game-like characteristics of VR experiences may create positive impressions that aren't dependent on extended engagement, consistent with research showing superior patient engagement in VR-based interventions (5, 9).

These methodological lessons complement our primary study findings, which demonstrated that VR rehabilitation using the same MDR-certified system achieved high patient satisfaction (mean USEQ 25.0 points) with excellent individual item performance, particularly for information clarity (4.63 ± 0.96) and perceived rehabilitation benefit (4.37 ± 1.12) (11). The present secondary analysis reveals that future studies examining dose-response relationships must address the fundamental methodological challenges of adequate statistical power and control for confounding factors to build upon these promising initial satisfaction outcomes. Our observation that session duration showed stronger correlations with specific satisfaction domains, particularly perceived rehabilitation benefit, aligns with research by Merians et al. (12) who noted that adequate session duration allows patients to experience therapeutic progression and mastery within VR environments. Similarly, the absence of correlation between cumulative VR time and overall satisfaction might support the “quality over quantity” principle discussed by Kim et al. (13), who emphasized that VR effectiveness depends more on appropriate task selection and individual adaptation than total exposure time. However, these interpretations remain speculative given our study's methodological limitations. Our mean session duration of 12.8 ± 4.5 min reflects individualized protocols based on patient tolerance in the subacute stroke phase. This differs from studies in chronic stroke populations that often employ longer fixed-duration sessions (30–60 min). However, shorter sessions may be more appropriate for subacute patients who frequently experience fatigue, attention deficits, and reduced tolerance for prolonged activities. While some studies suggest minimum session durations for motor learning benefits, our patient population's post-acute neurological status required flexible, tolerance-based approach rather than fixed minimum durations. Individual patient factors such as technological familiarity, cognitive capacity, and personal preferences may have greater influence on satisfaction than dosage parameters. This aligns with research demonstrating that VR's unique characteristics—real-time feedback, immersive environments, and task-specific training—can achieve therapeutic benefits through mechanisms different from conventional dose-dependent therapies (6, 7). While systematic reviews have advocated for flexible VR implementation approaches (1, 14), our study doesn't provide the rigorous evidence needed to guide such decisions.

Future research needs randomized controlled trials where VR dosage is assigned by protocol rather than determined by clinical circumstances. Based on our power calculations, such studies need at least 85–200 patients to detect clinically meaningful correlations with adequate statistical power. They should control for stroke severity and other baseline factors, examine multiple outcomes beyond satisfaction, and potentially include stratified analyses to identify patient subgroups who might benefit from different dosage approaches, as suggested by principles of distributed practice in motor learning research (8, 15).

## Study limitations

This study has several limitations that limit the interpretability of our findings. Most importantly, our sample size (*n* = 19) provided severely inadequate statistical power to detect clinically meaningful dose-response relationships. *Post-hoc* power analysis demonstrates that we had only 20% power to detect small-to-medium correlations (*r* = 0.1–0.3), which are often clinically significant in rehabilitation research. The absence of statistically significant correlations therefore cannot be interpreted as evidence against dose-response relationships.

Second, the strong correlation between VR sessions and hospital length of stay (*r* = 0.664) indicates substantial confounding by clinical factors, likely including stroke severity. VR dosage was not randomized but determined by clinical factors including medical stability, tolerance, and hospital stay duration. This creates a fundamental confounding problem where patients receiving more VR sessions may differ systematically in stroke severity, making our simple correlation analysis inappropriate for causal inference.

Third, the highly uneven distribution of VR exposure (58% of patients received ≤2 sessions) prevents meaningful analysis of dose-response thresholds. The data cannot inform clinical recommendations about optimal VR dosage because most patients clustered at very low exposure levels.

Given these substantial limitations, our findings must be interpreted as hypothesis-generating observations from an underpowered pilot study. A true dose-response relationship may well exist but remain undetected in this small, confounded sample.

## Conclusions

With only 19 patients, our study was too small to detect meaningful relationships between VR dosage and satisfaction—we had less than 25% power for clinically relevant effect sizes. The fact that patients with longer hospital stays got more VR sessions (*r* = 0.664) suggests our “dose” was really just a marker of stroke severity, not a treatment parameter we could evaluate. We can't recommend optimal VR dosing based on these results. Future studies need larger samples (at least 85 patients) and proper randomization of VR exposure to answer this question reliably.

## Data Availability

The original contributions presented in the study are included in the article/Supplementary Material, further inquiries can be directed to the corresponding author.
